# Inositols and Bone Health: Potential Therapeutic Applications in Osteoporosis Prevention and Treatment

**DOI:** 10.3390/nu17121999

**Published:** 2025-06-13

**Authors:** Fiammetta Cipriani, Lucio Gnessi, Mikiko Watanabe, Roberto Baldelli

**Affiliations:** 1 Section of Medical Pathophysiology, Food Science and Endocrinology, Department of Experimental Medicine, Sapienza University of Rome, 00161 Rome, Italy; 2 Endocrinology Unit, Department of Oncology and Medical Specialties, A.O. San Camillo-Forlanini, 00152 Rome, Italy

**Keywords:** bone remodeling, osteopenia, bone fractures, nutraceuticals, food supplements

## Abstract

The mechanisms underlying the disruption of bone balance are well known. To date, several possible treatments exist for osteoporosis, mostly based on inhibition of bone resorption. However, as osteoporosis is a disease that causes significant fragility, it merits a proactive prevention-based approach, which identifies risk factors, such as nutritional deficiencies, during the preosteoporotic stage. In this context, nutraceuticals may find application in delaying the onset of osteoporosis and prior to the need for pharmaceutical invention. The beneficial effects of inositol supplementation have been extensively studied in endocrinology and gynecology; herein, we discuss the potential of inositols in the prevention of osteoporosis, highlighting the link with bone metabolism and possible future applications.

## 1. Introduction

Osteoporosis is a condition characterized by low bone mineral density (BMD) and the microarchitectural deterioration of bone tissue, resulting in increased fragility [1]. Widely prevalent in postmenopausal women, osteoporosis must be screened in high-risk patient groups, including people undergoing long-term high-dose corticosteroid therapy, patients with comorbidities that increase fracture risk, and those receiving hormone-blocking therapy [2]. Peak bone density is generally achieved during an individual’s mid-twenties for bones like the spine and hip, whereas bones such as the radius reach peak density closer to age 40 [3]. After this period, bone density begins to decline, and fracture rates increase with age. Treatments aimed at reducing bone frailty can decrease hospitalizations and healthcare costs, significantly improving long-term quality of life [4].

The process that characterizes bone remodeling is primarily regulated by two cellular mechanisms, bone matrix neoformation, and bone resorption, which are controlled by osteoblasts and osteoclasts, respectively [5]. Both functions are tightly regulated to maintain a constant equilibrium in bone mass. Osteoclasts originate from hematopoietic precursors shared with the monocyte–macrophage lineage and require RANKL, a key mediator of the TNF family, for differentiation into activated multinucleated cells [6]. RANKL, produced by osteoblasts, bone marrow stromal cells, and lymphocytes, binds to its receptor RANK on osteoclast precursors and mature cells. The RANKL/RANK interaction turns on transcription factors through pathways that include PI3K (Phosphatidyl Inositol 3-Kinase)–Akt, MAPK, and NF-κB. In particular, the activation of TRAF6 induces NFATc1 and transcription of osteoclast-specific genes [7].

Osteoclasts interact with osteoblasts through factors like TGF-β and release microRNAs, such as miR-214-3p, to promote osteoclastogenesis via the PI3K/Akt pathway. Osteoblasts are regulated by WNT/β-catenin and osteoprotegerin (OPG), which inhibits osteoclastogenesis. They release vesicles containing RANKL and microRNAs to maintain bone balance. The PI3K–Akt pathway is crucial for both osteoblast and osteoclast functions, supporting survival, differentiation, and resorption, while integrin α5β1 aids osteoblast adhesion and matrix deposition [8].

Any disruption in this balance, particularly when bone resorption exceeds bone formation, can lead to bone diseases, with osteoporosis being the most common outcome [9]. Osteoporosis is a gradual condition characterized by the degradation of bone tissue. The structural integrity of bone is assessed using the T-score grading system, where a T-score above −1 indicates healthy bone, while a score below −2.5 indicates osteoporosis. Patients with a T-score between −1 and −2.5 are classified as having osteopenia, a condition where bone integrity is compromised but not to the extent of osteoporosis. Although osteopenia is considered a subclinical stage of osteoporosis, it is crucial to highlight that individuals with osteopenia have a higher incidence of fragility fractures compared to those with a T-score > −1 [10].

For both osteoporosis and osteopenia numerous therapies and preventative matters are described within the literature. Pharmacotherapeutic options include calcitonin, bisphosphonates, SERMs (e.g., raloxifene), and monoclonal antibodies [11]. Detailed discussion about these is beyond the scope of the present review. Lifestyle interventions, including physical activity and nutrition, are also essential in preventing and treating osteopenia and osteoporosis. Exercise promotes bone formation by inducing mechanical stress and improving hormonal and cytokine pathways [12]. Dietary calcium and vitamin D supplementation has traditionally prevented bone loss, as calcium is critical for bone homeostasis, and vitamin D enhances calcium absorption, reducing fracture risk [13]. However, recent evidence questions the efficacy of vitamin D alone, while its combination with calcium offers a modest reduction in fracture risk [14,15]. Other factors, such as vitamin K, are being investigated, as the low intake and systemic levels of vitamin K are associated with increased fracture risk [16,17]. A meta-analysis demonstrated an inverse relationship between dietary vitamin K intake and fracture risk [18], though its supplementation’s role in reducing osteoporosis remains uncertain [19]. Silicon intake has been linked to enhanced bone strength through increased collagen deposition [20]. Nutraceuticals, including resveratrol, polyphenols, and isoflavones, may further support bone health by improving BMD, inhibiting bone resorption, and enhancing osteoblast activity [21]. One such novel therapy being proposed is the use of inositols.

Inositol is a naturally occurring molecule that has been applied in the treatment of endocrinological conditions, such as polycystic ovary syndrome and gestational diabetes mellitus, due to its role as an insulin sensitizer [22]. Existing as a series of isomers, the most common of which are myo-inositol (MI) and D-chiro-inositol (DCI), inositol mediates osmoregulation, and it has been identified as an essential growth-promoting factor for mammalian cells [23]. MI is incorporated into eukaryotic cell membranes as phosphatidyl-myo-inositol, the precursor to inositol triphosphate (InsP3), which acts as a second messenger in the transduction of numerous endocrine signals, including FSH, TSH, and insulin [24]. MI is an organic osmolyte that regulates cellular responses to hypertonic environments; while it can be taken up by diffusion in highly concentrated environments, transport is primarily mediated by the sodium/myo-inositol cotransporter-1 (SMIT1) [25]. These MI transporters have been found in various tissues including kidney, brain, liver, pancreas, placenta, heart, and skeletal muscle [26]. Beyond playing a role in a variety of signaling pathways within human cells, inositol is also thought to play a role in the differentiation of osteoclasts. This review explores the emerging role of inositols and their metabolites as potential contributors to bone health.

## 2. Materials and Methods

A systematic review of the literature was conducted up to November 2024. To ensure a comprehensive and unbiased selection process, two independent researchers (FC and MW) performed the search across multiple databases, including PubMed, Cochrane Library, and Scopus. A predefined set of keywords and their synonyms was used to construct the search string: “Inositol” OR “Myo-inositol” OR “D-chiro-inositol” OR “Pinitol” AND “Osteoporosis” OR “Osteopenia” OR “Anti-resorptive therapies” OR “Osteogenesis”. The search was limited to the Title/Abstract fields and restricted to publications from the last 15 years (2009–2024).

In addition to database searches, the reference lists of all eligible articles were manually screened for further relevant studies.

The inclusion criteria were: (1) studies conducted on humans or rodents; (2) observational prospective or retrospective studies, cohort studies, and randomized controlled trials (RCTs); and (3) articles published in English. The exclusion criteria were: (1) studies not addressing the effects of inositols or their derivatives on bone metabolism or their role in cellular mechanisms such as osteoblastogenesis and osteoclastogenesis; (2) studies unrelated to the nutraceutical or metabolic applications of inositols; and (3) non-peer-reviewed materials, conference abstracts, and articles not available in English.

## 3. Results

**Figure 1:**
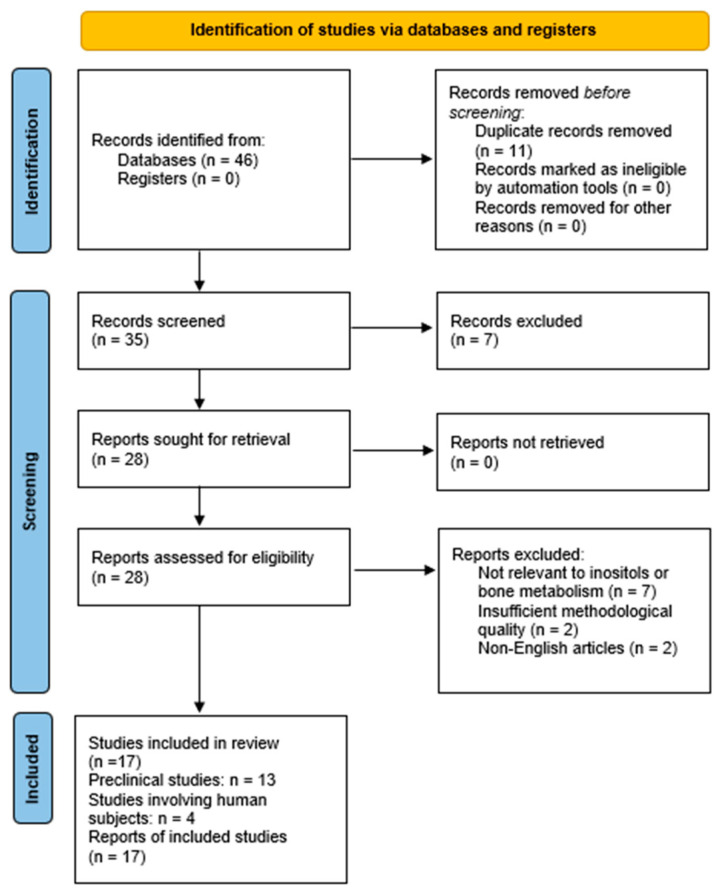
PRIMA flow diagram illustrating the study selection process.

A total of 46 studies were identified through database searches and manual review of reference lists. After removing 11 duplicates and excluding 7 studies based on titles and abstracts, 28 full-text articles were assessed for eligibility. Following the application of the inclusion and exclusion criteria, 17 articles were deemed eligible and were included in this review. These comprised 13 preclinical studies and 4 studies involving also human subjects (Table 1 and Figure 1). Of the four included human studies, only two provided sufficient data to allow inclusion in a quantitative synthesis; however, a formal meta-analysis was not performed, as these studies lacked comparable outcome metrics—such as mean changes in BMD or consistent measures of variance across defined exposure groups—limiting the interpretability and clinical relevance of a pooled estimate.

### Inositols and Their Role in Bone Metabolism

Animal studies investigating the role of MI in bone metabolism have shown that inositol deficiency reduced bone mineralization and its supplementation positively modulated the balance between osteoblasts and osteoclasts. The observation of SMIT1 knockout mice showed that SMIT1 deficiency caused adverse effects on prenatal bone mass and postnatal bone remodeling. Furthermore, the supplementation of MI in the diet after weaning partially corrected bone defects in adult mice, and improved bone structure in wild-type animals [27].

In humans, MI is consumed partially through the diet, typically 1 g/day largely through the consumption of cereals, legumes, oilseeds, and nuts; however, a significant percentage, 4 g/day, of the daily requirement is synthesized endogenously within the kidneys [28]. Western diets are high in fat and sugar and very often low in fiber and key nutrients such as inositol and inositol hexaphosphate (phytates). The reduced bioavailability of inositol in living organisms can be due to several factors including poor diet, reduced intestinal absorption, the deregulation of metabolism, and increased excretion [29]. High-sucrose diets, in addition to conditions characterized by altered glucose metabolism, impair inositol availability and increase its degradation, inhibiting both the biosynthesis and absorption of MI. Several studies highlight the significance of the contribution of inositols in the development of many diseases; however, limited research has been published to date explaining how and why pathological inositol deficiency might occur and how this may affect health.

Inositol phosphates are found within the cell in two distinct forms, either as membrane-anchored phosphatidylinositol (PI) or as water-soluble PI. Both forms of PI act as second messengers in signal transduction pathways, mediating protein phosphorylation and participating in chromatin remodeling and gene expression, while also facilitating mRNA transition from the nucleus [30]. Some of these phosphate metabolites and the enzymes involved in inositol metabolism may play key roles in human health.

One notable Inositol phosphate is Inositol polyphosphate 4 phosphatase type IIa (Inpp4ba), a member of the PI3-kinase signaling pathway. Ex vivo and in vivo analyses have demonstrated that Inpp4b is a modulator of osteoclast differentiation, able to inhibit osteoclastogenesis via the nuclear factor of the activated T cells c1 (Nfatc1) signaling pathway [31]. Consequently, mice lacking INPP4B suffer from bone loss and osteoporosis, as INPP4Bα acts on intracellular calcium to inhibit the nuclear localization of NFATc1 and subsequently the transcription of osteoclast-specific target genes. INPP4B maps to chromosome 4q where quantitative loci for BMD have been located, suggesting it as a candidate gene for BMD variability in humans [32].

Myo-inositol hexaphosphate (InsP6 or phytate) is present in human organs and tissues in its ionized form. Phytate is of interest to bone health as it may act as a potent inhibitor of calcium salt crystallization, binding to crystal surfaces in a similar fashion to other polyphosphates, pyrophosphate, and bisphosphonates [33]. IP6 inhibits the PI3K-Akt signaling pathway, reducing cell proliferation, survival and angiogenesis. By decreasing the phosphorylation of Akt and GSK-3 (glycogen synthase kinase-3) and suppressing vascular endothelial growth factor and nitric oxide synthase, IP6 indirectly influences bone remodeling. These results suggest that IP6 is a promising therapeutic agent for cancer and a potential modulator of bone health [34].

In vitro studies have shown that phytate effectively inhibits osteoclastogenesis in both RAW 264.7 murine monocyte/macrophage cells and human primary osteoclasts [35]. These findings suggest that IP6 could protect against osteoporosis by suppressing osteoclast activity and preventing hydroxyapatite dissolution. Furthermore, IP6 demonstrates cell-type-specific effects on osteoblast differentiation: it enhances alkaline phosphatase (ALP) expression in human umbilical cord mesenchymal stem cells (hUC-MSCs) but reduces osteoblast marker expression in MC3T3-E1 pre-osteoblasts. These dual actions highlight the potential of IP6 as a therapeutic agent for managing osteoporosis by simultaneously regulating bone resorption and formation [36].

The study by Sanchis et al. [35] investigated the association between phytate intake and BMD in Mediterranean postmenopausal women, finding that higher phytate intake was linked to better BMD. This suggests that phytate, found in foods like legumes, whole grains, and nuts, may play a role in supporting bone health in postmenopausal women [37]. Furthermore, a cohort study indicated that participants with high urinary phytate concentrations experienced reduced bone mass loss over a 12-month period, reinforcing the idea of a positive link between phytate consumption and bone density [38]. These findings imply that low phytate intake may be associated with an increased risk of reduced bone mass, further highlighting the potential protective role of phytate in maintaining bone health [39]. Works by Yoshiko, Vucenik, and colleagues offer a comprehensive analysis of the potential of IP6 in promoting bone health. Phytate’s ability to inhibit the crystallization of calcium salts by binding to calcium crystal surfaces, akin to the action of bisphosphonates and pyrophosphates, suggests it could play a crucial role in regulating BMD and preventing excessive bone resorption [40]. Moreover, Vucenik, and Druzijanic [41] explore the broader biological effects of IP6, particularly its anticancer properties, highlighting its role in reducing oxidative stress, a key factor in both bone degradation and cancer progression. Their findings support the hypothesis that phytate, through its antioxidant and mineral-regulating actions, may offer a multi-faceted therapeutic benefit.

For many years, phytate has been considered an antinutrient, due to its high affinity for various ions including magnesium, zinc, calcium, and iron [42]. This effect can be reversed by phytase-induced phytic acid degradation during food processing (via phytases present in the plant/flour) and during digestion (via phytase activity expressed in the microbiota residing in the intestinal tract) [43]. The number of phosphate groups bound to inositol plays a crucial role in determining the inhibitory effects of phytates on mineral absorption. When inositol reaches a higher degree of phosphorylation (InsP5, InsP6), there is significant inhibition of calcium and zinc absorption; however, during fermentation, microbial enzymes break down phytates into their less phosphorylated forms (InsP3, InsP4) [44]. Inositol pyrophosphates (InsP7 and InsP8), which possess even higher phosphorylation than phytates (InsP5, InsP6), have critical roles in metabolic regulation. These molecules modulate phosphate metabolism, energy balance, and intracellular signaling pathways [45]. Nagpal highlights their ability to act as phosphate donors, influencing cellular ATP turnover and regulating phosphate storage and release. Their distinct properties allow them to integrate energy sensing and phosphate homeostasis, which could explain their potential effects on nutrient absorption and bone health when interacting with lower phosphorylated inositol forms (InsP3, InsP4) [46]. In bone marrow, adipocytes and osteocytes, are derived from bone marrow-derived stem/stromal mesenchymal cells (BMMSCs). In cases of elder or obese individuals, changes can occur within bone marrow, with increased adipogenesis over osteogenesis leading to skeletal involution and marrow adiposity [47]. It is therefore vital to understand the process and contributing factors of adipogenesis within bone marrow when considering effective osteoporosis treatment. In this context, Inositol hexakisphosphate kinase 1 (IP6K1) has been considered a potential target in treating obesity, metabolic diseases, and more recently bone disorders [48]. In MSCs isolated from mice, Boregowda et al. demonstrated that IP6K1 knockout BMMSCs reduced adipocyte differentiation and increased osteocyte differentiation [49]. Moreover, in models of diet-induced obesity (DIO) mice, daily administration of IP6K1 inhibitors offered protection against high-fat-diet-induced metabolic disturbances, resulting in a preservation of bone density [50]. Insights from recent studies indicate that IP6K1 plays a pivotal role in regulating metabolic processes such as insulin secretion and obesity. This highlights its potential as a therapeutic target for addressing these interconnected disorders by modulating energy homeostasis and nutrient-sensing pathways [51].

Additionally, IP6K2 (inositol hexakisphosphate kinase 2), another enzyme within the inositol pyrophosphate pathway, has been shown to regulate InsP7 metabolism. This molecule is critical for maintaining phosphate homeostasis and energy balance, which are essential for skeletal health under metabolic stress [52].

D-pinitol (3-O-methyl-D-chiro-inositol) is a methylated analog of DCI and it is actively converted to DCI within the acidic conditions of the stomach [53]. Unlike MI, which is readily available through the diet, few foods, namely buckwheat, soy lecithin, carob, and lentils, contain DCI in significant levels [54]. It is therefore difficult to consume sufficient amounts of DCI, which is indeed mainly synthesized in the body from MI [26].

Previous studies have highlighted the osteo-protective action of D-pinitol which seems to play an anti-osteoclastogenic role. Yu et al. concluded that D-pinitol and DCI act as inhibitors of NkF-B/RANKL-dependent osteoclast differentiation, via the down-regulation of NFATc1 and by the inhibition of NFk-B in vitro [55]. However, this mechanism is up for debate as, according to another study by Liu et al., D-pinitol inhibits osteoclastogenesis through diminished NF-KB activation [56]. Moreover, Liu et al. administered estrogen or pinitol in osteoporosis mouse models obtained via ovariectomy, with both treatments increasing the femoral content of calcium and phosphorus, and D-pinitol treatment improving the osteoporotic status of the mice. Of note, D-pinitol appears to have no effect on osteoblast function, demonstrating a specific action on osteoclastic mechanisms. Moreover, ovariectomy causes a statistically significant decrease in serum DCI, which is recovered in serum and bone, indicating the osteo-protective action of pinitol is achieved via the conversion of pinitol to DCI [57]. D-pinitol also exhibits an anti-hyperglycemic effect, which would be expected given the insulin-sensitizing effect of DCI. Interestingly, bone mass loss induced by diabetes-related osteoporosis is recovered through D-pinitol supplementation, suggesting the mechanism of action is partially reliant on the improvement of metabolic functions [58]. Inositols, particularly MIand DCI, show significant promise as nutraceuticals in preventing and treating osteoporosis. They exhibit osteoprotective properties through various molecular pathways that regulate bone metabolism, contributing to improved BMD and overall skeletal health by preserving bone microarchitecture, as suggested by preclinical studies.

**Table 1:** Summary of key findings from the 17 studies included in the review, categorized by experimental design (human or preclinical).

Author	Study Design	Primary Outcome	Sample Size or Study Model	Main Results
Sanchis et al. [35]	Human	Changes in BMD after phytate intake	*n* = 440 women	Higher phytate intake associated with higher BMD
López-González et al. [38]	Human	Changes in BMD after phytate intake	*n* = 157 postmenopausal women	Positive correlation between phytate intake and BMD
Gonzalez et al. [39]	Human	Changes in bone mass loss and urinary phytate	*n* = 212 women	Higher urinary phytate linked to reduced bone loss
Navarro et al. [53]	Human	Hormonal response to D-pinitol	*n* = 25 healthy volunteers	D-pinitol altered endocrine markers
Sanchis et al. [33]	Preclinical—in vitro	Effect of IP6 on osteoclast activity	RAW264.7, human cell cultures	IP6 inhibited osteoclastogenesis
Arriero et al. [36]	Preclinical—in vitro	ALP expression and osteoblast differentiation	hUC-MSCs, MC3T3-E1 cells	Differential regulation of osteoblast markers
Arriero et al. [37]	Preclinical—in vitro	IP6 inhibition of osteoclastogenesis	RAW264.7, human primary osteoclasts	Inhibition of osteoclast differentiation
Yu et al. [55]	Preclinical—in vitro	DCI/D-pinitol and NFATc1 in osteoclasts	Murine preosteoclasts	Inhibition of osteoclastogenesis via NFATc1
Liu et al. [56]	Preclinical—in vitro	RANKL-induced osteoclastogenesis and pinitol	RAW264.7 cells	D-pinitol inhibited RANKL pathway
Gu et al. [34]	Preclinical—in vitro	IP6 effects on PI3K–Akt signaling	Prostate carcinoma cells	Inhibition of signaling and proliferation
Dai et al. [27]	Preclinical—in vivo	MI supplementation in SMIT1 KO mice	KO and WT mice	Partial rescue of bone phenotype
Boregowda et al. [49]	Preclinical—in vitro and in vivo	IP6K1 inhibitor effects on bone	MSC cultures; WT and Ip6k1^−^/^−^ mice	Preserved BMD and reduced bone loss
Boregowda et al. [50]	Preclinical—in vitro and in vivo	IP6K1 inhibitor effects on bone	Human MSCs; C57BL/6 mice	Preserved BMD and reduced bone loss
Liu et al. [57]	Preclinical—in vivo	Pinitol treatment in OVX mice	OVX mice	Improved BMD with pinitol
Liu & Koyama [58]	Preclinical—in vivo	Pinitol in diabetic osteoporosis	Diabetic mice	Restored bone loss with metabolic improvement
Ferron et al. [32]	Preclinical—in vivo	INPP4B gene and bone phenotype	INPP4B KO mice	KO mice showed bone loss
Ito M et al. [51]	Preclinical—in vivo	The role of IP6K2/IP7 in the enteric nervous system	WT and IP6K2-KO C57BL/6 mice	IP6K2 regulates IP7 metabolism, affecting enteric neuronal activity and gut motility

**Abbreviations:** ALP (Alkaline Phosphatase), BMD (Bone Mineral Density), CCM (Complete Culture Media), DCI (D-Chiro-Inositol), hUC-MSCs (Human Umbilical Cord Mesenchymal Stem Cells), IP6 (Inositol Hexakisphosphate), IP6K1 (Inositol Hexakisphosphate Kinase 1), INPP4B (Inositol Polyphosphate-4-Phosphatase Type II B), KO (Knockout), MC3T3-E1 (Murine Calvarial Preosteoblast Cell Line), MI (Myo-Inositol), MSC (Mesenchymal Stem Cell), NFATc1 (Nuclear Factor of Activated T-Cells, Cytoplasmic 1), OVX (Ovariectomized), PI3K–Akt (Phosphoinositide 3-Kinase––Protein Kinase B Pathway), RAW264.7 (Murine Macrophage Cell Line), RANKL (Receptor Activator of Nuclear Factor κB Ligand), and WT (Wild Type).

MI enhances osteoblast function and inhibits osteoclast differentiation via RANKL-mediated pathways, while DCI and its derivative D-pinitol reduce osteoclast activity, particularly in conditions like postmenopausal osteoporosis and diabetes-related bone loss. Clinical observations associate inositol deficiency with lower BMD and poor bone health. Supplementation with inositols, including phytates, has been linked to improved bone quality, reduced bone mass loss, and slower rates of BMD loss, especially in populations at high risk for osteoporosis, such as postmenopausal women.

Integrating inositols into preventive strategies and treatment regimens could offer a novel approach to enhancing bone density and reducing fracture risk. Their possible extends to osteopenia management, potentially delaying the onset of osteoporosis, and they could serve as part of an integrative treatment alongside pharmaceuticals for osteoporotic patients. Furthermore, inositols may have potential for treating bone disorders in breast and prostate cancer patients who have undergone hormonal and/or chemotherapy. These therapies are known to cause a premature reduction in BMD [59]; consequently, calcium and vitamin D supplementation is recommended in addition to antiresorptive treatment [60]. Inositol’s may be employed with the current guidelines due to its anti-osteoclastic action, and merits further study. Given their anti-osteoclastic action, inositols could complement current osteoporosis guidelines, including calcium, vitamin D, and antiresorptive therapies like bisphosphonates or denosumab.

**Figure 2:**
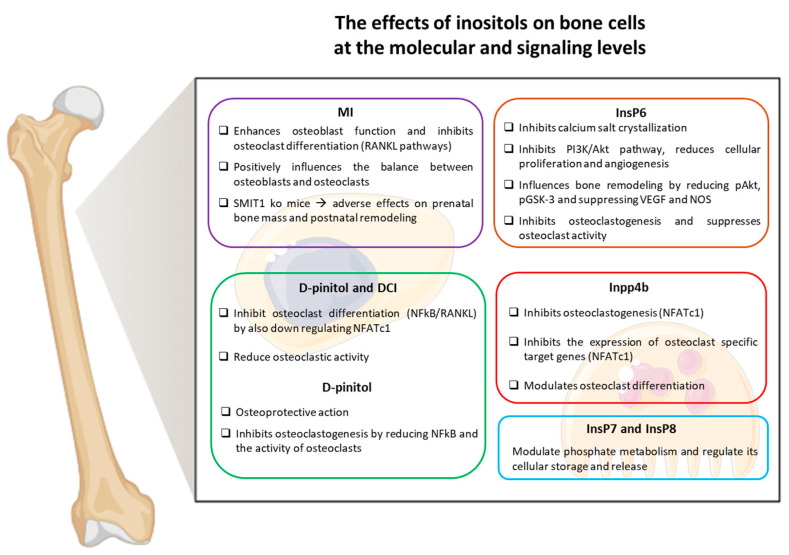
Graphical representation of the effects of inositols on bone cells at the molecular and signaling levels. The figure summarizes the principal pathways on osteoblasts and osteoclasts by highlighting the activity and the effect of different types of inositol. Figure prepared in Biorender™.

Despite promising preclinical data, clinical trials are essential to confirm the efficacy of inositol supplementation in reducing fracture risk and improving BMD in humans. Additionally, understanding how inositols interact with dietary interventions and existing pharmacological treatments could clarify their role in osteoporosis management. As skepticism grows around traditional bone health supplements, inositols represent an exciting research avenue, with the potential to revolutionize the prevention and treatment of osteoporosis. A graphical summary of the molecular mechanisms by which different inositol isomers influence bone cells is presented in Figure 2.

## 4. Conclusions

Inositols hold significant promise as a therapeutic option complementary to the current strategies for bone health, particularly in the settings of prevention and early bone loss such as osteopenia. However, they should not be regarded as a substitute for established pharmacologic therapies in the treatment of osteoporosis. Their integration into clinical practice must be guided by future research aimed at elucidating their precise mechanisms of action, effectiveness, and safety. As we advance our understanding of the molecular mechanisms underlying bone remodeling, inositols may emerge as a key component in the fight against osteoporosis and related bone disorders, offering hope for improved outcomes in at-risk populations.
